# Radiomics of diffusion-weighted MRI compared to conventional measurement of apparent diffusion-coefficient for differentiation between benign and malignant soft tissue tumors

**DOI:** 10.1038/s41598-021-94826-w

**Published:** 2021-07-27

**Authors:** Seung Eun Lee, Joon-Yong Jung, Yoonho Nam, So-Yeon Lee, Hyerim Park, Seung-Han Shin, Yang-Guk Chung, Chan-Kwon Jung

**Affiliations:** 1 Department of Radiology, Seoul St. Mary’s Hospital, College of Medicine, The Catholic University of Korea, 222 Banpo-daero, Seocho-gu, Seoul, 06591 Republic of Korea; 2 Division of Biomedical Engineering, Hankuk University of Foreign Studies, Yongin-si, Gyeonggi‐do Republic of Korea; 3 Department of Radiology, Soonchunhyang University Cheonan Hospital, Dongnam-gu, Cheonan, Chungcheongnam-do Republic of Korea; 4 Department of Orthopedic Surgery, College of Medicine, The Catholic University of Korea, Seoul, Republic of Korea; 5 Department of Pathology, College of Medicine, The Catholic University of Korea, Seoul, Republic of Korea

**Keywords:** Sarcoma, Machine learning, Musculoskeletal system

## Abstract

Diffusion-weighted imaging (DWI) is proven useful to differentiate benign and malignant soft tissue tumors (STTs). Radiomics utilizing a vast array of extracted imaging features has a potential to uncover disease characteristics. We aim to assess radiomics using DWI can outperform the conventional DWI for STT differentiation. In 151 patients with 80 benign and 71 malignant tumors, ADC_mean_ and ADC_min_ were measured on solid portion within the mass by two different readers. For radiomics approach, tumors were segmented and 100 original radiomic features were extracted on ADC map. Eight radiomics models were built with training set (n = 105), using combinations of 2 different algorithms—multivariate logistic regression (MLR) and random forest (RF)—and 4 different inputs: radiomics features (R), R + ADC_min_ (I), R + ADC_mean_ (E), R + ADC_min_ and ADC_mean_ (A). All models were validated with test set (n = 46), and AUCs of ADC_mean_, ADC_min_, MLR-R, RF-R, MLR-I, RF-I, MLR-E, RF-E, MLR-A and RF-A models were 0.729, 0.753 0.698, 0.700, 0.773, 0.807, 0.762, 0.744, 0.773 and 0.807, respectively, without statistically significant difference. In conclusion, radiomics approach did not add diagnostic value to conventional ADC measurement for differentiating benign and malignant STTs.

## Introduction

DWI is a functional MRI that provides unique information about the microarchitecture of tissue and how it affects the diffusion of water molecules. In musculoskeletal field, it is considered to improve the detection of tumorous or infectious lesions, differentiation of benign and malignant tumors, and evaluation of treatment response when combined with standard MRI. DWI is advantageous especially in the case of tumor characterization because it can provide the quantitative information with ADC map. It can also give additional information for determining various extracellular component such as hemorrhage, mineralization, fat and myxoid tissue^1,2^.

Radiomics is an emerging field of study that utilizes the vast arrays of quantitative features extracted from volumetric image, which is comprised of millions of voxels^3^. Typical radiomic assessment includes analysis of texture, shape, and size; and, the acquired features hold information about tumor pathophysiology, which can be harnessed for diagnosis, prognosis and therapeutic modality in clinical field.

In musculoskeletal radiology, differentiation of the malignant soft tissue sarcomas from benign tumors is important for planning proper treatment. Due to their pathologic heterogeneity with various origin, location and overlapping imaging features on conventional MRI, standard MRI is frequently insufficient for differentiating soft tissue tumors. Several previous studies demonstrated increased diagnostic accuracy of malignant soft tissue sarcoma by obtaining additional DWI with standard MRI^4–6^. However, clinical utility of radiomics based on ADC map, regarding the diagnosis of soft tissue sarcoma, has not been studied yet. Therefore, we aim to assess the diagnostic performances of various radiomics models based on DWI and ADC map and to compare them with that of ADC_mean_ and ADC_min_ in this study.

## Result

### Patient demographics

Among the included 151 patients, 71 patients (47%) were diagnosed as malignant soft tissue sarcoma. Lesions with intermediate biologic behavior including desmoid type fibromatosis, fibrohistiocytic tumor, inflammatory myofibroblastic tumor and solitary fibrous tumor were considered as benign. Specific histological types and locations of included tumors are shown in Table 1. The average time interval between MRI and pathologic assessment was 28 days (range; 0–357 days). The proportion of malignant soft tissue tumor was 45% (47/105 cases) in training set and 52% (24/46 cases) in test set. There was no statistically significant difference of age, sex, ADC_mean_ and ADC_min_ values between the training and test sets. Baseline demographics and clinical characteristics of the patients are summarized in Supplementary Table (S1).

**Table 1:** Various histology of soft tissue sarcoma of the included patients.

Soft tissue tumors	
**Benign and borderline tumors (n = 80)**	
Histology	Schwannoma (n = 41), Tenosynovial giant cell tumor (n = 11), Desmoid type fibromatosis (n = 8), Neurofibroma (n = 5), Myxoma (n = 3), Inflammatory myofibroblastic tumor (n = 2), Solitary fibrous tumor (n = 1), Nodular fasciitis (n = 1), Fibrohistiocytic tumor (n = 1), Spiradenoma (n = 1), Granular cell tumor (n = 1), Angiofibroma (n = 1), Angioleiomyoma (n = 1)
Location	Neck (n = 2), Shoulder and axilla (n = 8), Upper extremity (n = 17), Hand and Wrist (n = 17), Trunk (n = 6), Hip and Groin (n = 7), Lower extremity (n = 21), Foot and Ankle (n = 2)
**Malignant tumor (n = 71)**	
Histology	Undifferentiated pleomorphic sarcoma (n = 18), Myxoid liposarcoma (n = 12), Myxofibrosarcoma (n = 11), Synovial sarcoma (n = 7), Malignant peripheral nerve sheath tumor (n = 6), Rhabdomyosarcoma (n = 4), Leiomyosarcoma (n = 4), Dedifferentiated liposarcoma (n = 2), Pleomorphic liposarcoma (n = 1), Alveolar soft-part sarcoma (n = 1), Angiosarcoma (n = 1), Epithelioid sarcoma (n = 1), Extraskeletal myxoid chondrosarcoma (n = 1), Low grade fibromyxoid sarcoma (n = 1), Malignant solitary fibrous tumor (n = 1)
Location	Shoulder and axilla (n = 1), Upper extremity (n = 10), Hand and Wrist (n = 2), Trunk (n = 6), Hip and Groin (n = 16), Lower extremity (n = 34), Foot and Ankle (n = 2)

### Diagnostic performance of ADC value

**Figure 1:**
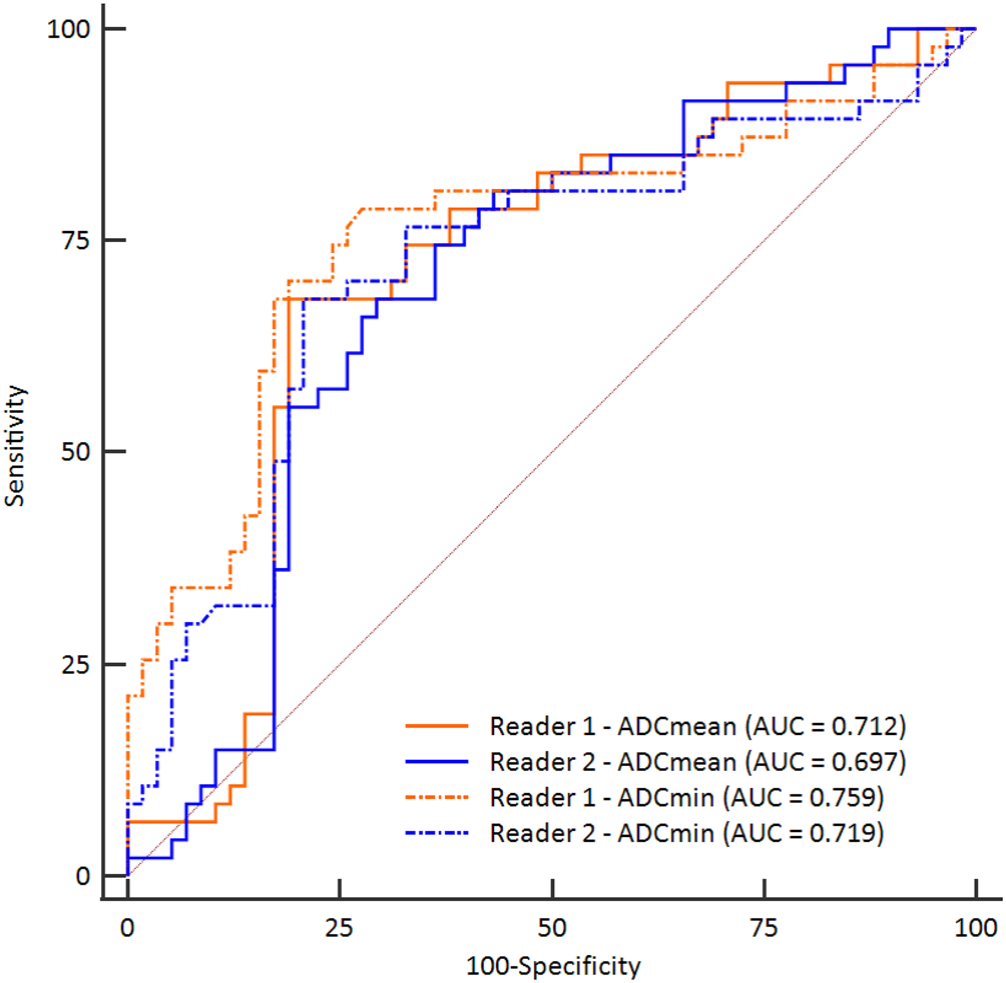
ROC curves of ADC_mean_ and ADC_min_ of reader 1 and reader 2 in training set. The plots were generated using MedCalc for Windows, version 19.0 (MedCalc Software, Ostend, Belgium).

The mean ROI size of all tumors was 62.5 mm^2^ (range; 17.5–133.9 mm^2^) and 69.5mm^2^ (range; 15.4–145.6 mm^2^) in both readers, respectively. The interobserver agreement of ADC values in ROIs was excellent between 2 readers : intraclass correlation coefficients of ADC_mean_ = 0.97 and ADC_min_ = 0.93. With the ADC values assessment, the average and standard deviation of ADC_mean_ and ADC_min_ value in regions of interest (ROI) in training set (n = 105) were 1448.59 ± 567.98, 1015.55 ± 492.67 in reader 1 and 1429.26 ± 554.75, 959.07 ± 480.27 in reader 2, respectively (Supplementary Table 1). Specifically, the average and standard deviation of ADC_mean_ and ADC_min_ were 1199.69 ± 443.41, 788.32 ± 463.59 in reader 1 and 1215.44 ± 445.90, 776.74 ± 465.70 in reader 2 in malignant soft tissue tumors, and 1632.90 ± 576.41, 1221.14 ± 478.52 in reader 1 and 1602.53 ± 573.52, 1106.83 ± 439.37 in reader 2 in benign soft tissue tumors, respectively. Both ADC_mean_ and ADC_min_ values were significantly associated with malignant soft tissue tumor differentiation in reader 1 and 2 (*p* value < 0.001). The receiver operating characteristic (ROC) curves are demonstrated in Fig. 1; and AUCs of ADC_mean_ and ADC_min_ were 0.712 and 0.759 in reader 1, and 0.697 and 0.719 in reader 2, respectively.

### Reproducibility of segmentation and influence of normalization methods on feature selection

Among randomly selected 20 tumor images for evaluation of segmentation reproducibility, 13 tumors (65%) were malignant. The average and standard deviation of Dice coefficient between 3 different segmentations by 3 readers were 0.92 ± 0.07 (range from 0.57 to 1).

Radiomic features were extracted, and top 20 radiomic features were selected by univariate regression test for each normalization method. When top 20 selected features were compared, 7 features were commonly selected regardless of the normalization methods. Common radiomic features between two normalization methods were 55% (11/20 features), 70% (14/20 features) and 60% (12/20 features) in whole-image method, VOI method and VOI-dilation method, respectively (Table 2).

**Table 2:** Common features among the top 20 original radiomic features from each normalization method for malignant tumor prediction

Normalization method	Method 1^†^, 2^‡^ and 3*	Method 1 and 2	Method 1 and 3	Method 2 and 3
Common features	GLCM Joint entropy	Shape Least axis length	First order Interquartile range	GLCM Inverse variance
	GLCM Correlation	GLCM Cluster tendency		Shape Mesh volume
	GLCM Difference entropy	GLDM Dependence nonuniformity		GLCM Imc2
	First order 10 percentile			First order Maximum
	GLSZM Zone variance			
	Shape Sphericity			
	GLCM Cluster shade			

^†^Normalization method covering all voxels from whole image.

^‡^Normalization method covering the voxels from VOI only.

*Normalization method covering the voxels from VOI and its 2 mm-dilated circumference.

### Diagnostic performances of 8 classification models

The AUCs of MLR-R, MLR-I, MLR-E and MLR-A models with training set were 0.848, 0.883, 0.876 and 0.883 in reader 1 and 0.848, 0.873. 0.875 and 0.875 in reader 2, respectively. Also the AUCs of RF-R, RF-I, RF-E and RF-A models with training set were 0.807, 0.846, 0.841 and 0.860 in reader 1 and 0.807, 0.820, 0.834 and 0.841 in reader 2, respectively. All results including AUCs, sensitivity, specificity and accuracy of 8 built models were presented in Table 3. In MLR-A and RF-A models, ADC_min_ was selected as second-most important and the most important feature in reader 1, and ADC_mean_ was not included in model generation. In contrast, ADC_mean_ was selected as second-most important and fourth-most important feature in reader 2, and ADC_min_ was not included in MLR-A and RF-A model generation.

**Table 3:** Diagnostic performance of ADC_mean_, ADC_min_ and radiomics model in training set and test set.

			Single parameter		Radiomics model							
			ADC_mean_	ADC_min_	MLR-R	RF-R	MLR-I	RF-I	MLR-E	RF-E	MLR-A	RF-A
Train set	R1	AUC	0.712	0.759	0.848	0.807	0.883	0.846	0.876	0.841	0.883	0.860
		Sensitivity (%)	68.1	70.2	78.7	66.0	78.7	68.1	74.5	74.5	78.7	68.1
		Specificity (%)	81.0	81.0	77.6	79.3	84.5	84.5	86.2	82.8	84.5	86.2
		Accuracy (%)	70.5	74.3	78.1	73.3	81.9	77.1	81.0	79.0	81.9	78.1
	R2	AUC	0.697	0.719	0.848	0.807	0.873	0.820	0.875	0.834	0.875	0.841
		Sensitivity (%)	68.1	68.1	78.7	66.0	78.7	70.2	72.3	72.3	72.3	68.1
		Specificity (%)	70.7	79.3	77.6	79.3	82.8	79.3	87.8	81.0	82.8	82.8
		Accuracy (%)	69.5	74.3	78.1	73.3	81.0	75.2	78.1	77.1	78.1	76.2
Test set	R1	AUC	0.729	0.753	0.698	0.700	0.773	0.807	0.762	0.744	0.773	0.807
		*p* value^†^		0.551	0.745	0.782	0.456	0.259	0.537	0.827	0.456	0.259
		*p* value^‡^	0.551		0.559	0.599	0.653	0.408	0.861	0.907	0.653	0.408
		Sensitivity (%)	66.7	70.8	58.3	75.0	75.0	83.3	70.8	70.8	75.0	83.3
		Specificity (%)	81.8	86.4	68.2	63.6	63.6	59.1	54.5	59.1	63.6	59.1
		Accuracy (%)	73.9	78.3	63.0	69.6	69.6	71.1	63.0	65.2	69.6	71.1
	R2	AUC	0.689	0.711	0.698	0.700	0.727	0.771	0.742	0.775	0.742	0.775
		*p* value^†^		0.562	0.932	0.922	0.664	0.306	0.312	0.330	0.312	0.330
		*p* value^‡^	0.562		0.895	0.915	0.841	0.430	0.561	0.476	0.561	0.476
		Sensitivity (%)	54.2	62.5	58.3	75.0	70.8	66.7	75.0	70.8	75.0	70.8
		Specificity (%)	90.9	90.9	68.2	63.6	63.6	59.1	54.5	59.1	54.5	59.1
		Accuracy (%)	71.7	76.1	63.0	69.6	67.4	63.0	65.2	65.2	65.2	65.2

*R1* Reader 1, *R2* Reader 2.

^†^*p* value calculated from Delong test for comparison of diagnostic performance between radiomics model and ADC_mean_ in test set.

^‡^*p* value calculated from Delong test for comparison of diagnostic performance between radiomics model and ADC_min_ in test set.

**Figure 2:**
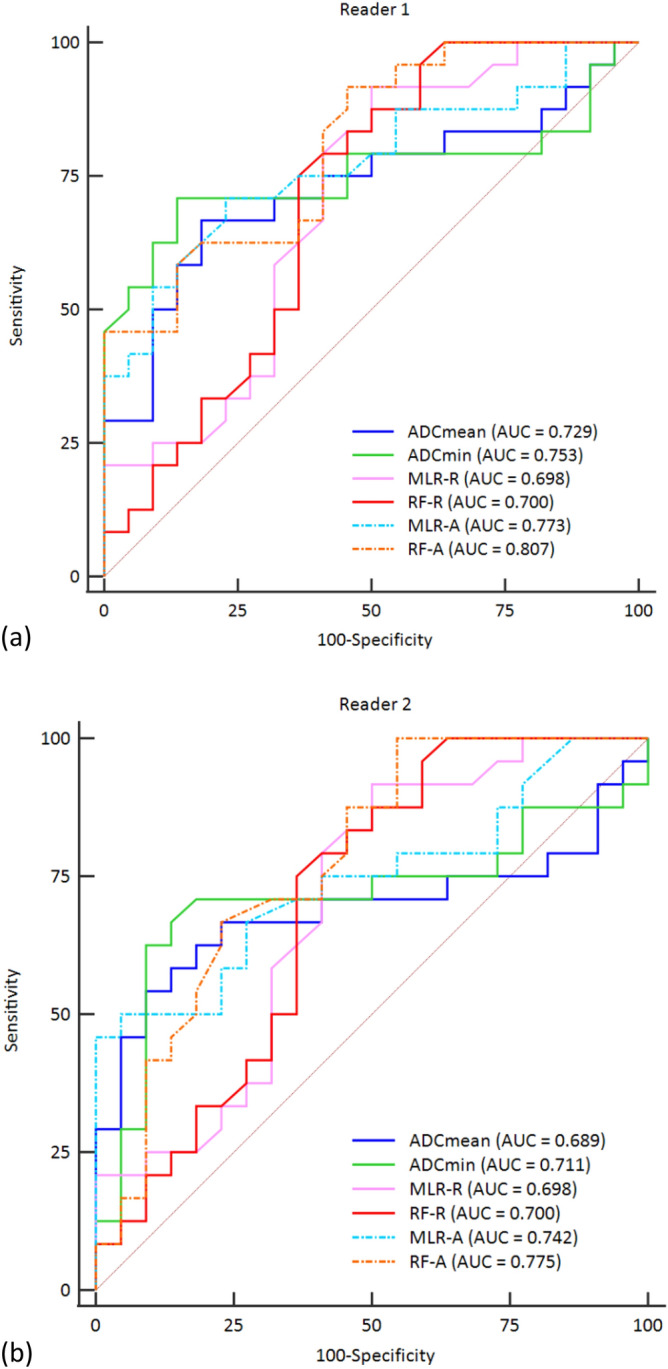
Comparison between the diagnostic performance of ADC values and 4 radiomics models (MLR-R, RF-R, MLR-A and RF-A) in reader 1 (**a**) and reader 2 (**b**). The plots were generated using MedCalc for Windows, version 19.0 (MedCalc Software, Ostend, Belgium).

The AUCs of ADC_mean_, ADC_min_, MLR-R, RF-R, MLR-I, RF-I, MLR-E, RF-E, MLR-A and RF-A radiomics models in test set were 0.729, 0.753, 0.698, 0.700, 0.773, 0.807, 0.762, 0.744, 0.773 and 0.807 in reader 1, and 0.689, 0.711, 0.698, 0.700, 0.727, 0.771, 0.742, 0.775, 0.742 and 0.775 in reader 2, respectively. The other results compared in test set are summarized in Table 3. Comparisons of AUCs between ADC_mean_, ADC_min_ and 4 radiomics models (MLR-R, RF-R, MLR-A, RF-A) did not show any significant difference in both readers (reader 1; *p* value = 0.745, 0.559 with MLR-R model, *p* value  = 0.782, 0.599 with RF-R model, *p* value  = 0.456, 0.653 with MLR-A model, *p* value  = 0.259, 0.408 with RF-A model, reader 2; *p* value  = 0.932, 0.895 with MLR-R model, *p* value  = 0.922, 0.915 with RF-R model, *p* value  = 0.312, 0.561 with MLR-A model, *p* value  = 0.330, 0.476 with RF-A model) (Fig. 2).

## Discussion

In this study, we compared the use of ADC and radiomics approach with ADC maps for differentiation of benign and malignant STT. Eight radiomics models estimated with the test set showed similar performances to ADC measurement. When the models using both ADC measurement and radiomics features were built with training set, ADC was included as highly ranked features and diagnostic performances were consistently higher than the single use of ADC in all radiomics models. However, there was no increment in diagnostic performances when the trained models were applied to the test set. The discrepant results between training and test sets may attribute to different composition in pathologic entities, different data collection periods, or just a random noise related to small sample size.

It is widely known that ADC map provides additional benefits to conventional MR images in differentiating soft tissue sarcoma from benign tumors^2,4,5,7^, with quantitative information of tumor cellularity. According to Lin et al., the ADC map is correlated pixelwise with histology in terms of extracellular space and nuclear size^8^. Also, there are several previous studies about repeatability of ADC values across institutions and MRI vendors^9–11^. Due to its quantifiable and reliable properties, the ADC map emerged as a target for radiomics analysis^12–14^. A recent study published by Peerling et al. showed substantial test–retest stability (25–29%) of ADC based features in radiomic feature analysis within multicenter and multi-vendor trial with patients of lung cancer, ovarian cancer and liver metastasis of colorectal cancer^13^. However, there has been no previous study about application of radiomics analysis using ADC map on the differentiation of malignant soft tissue tumors.

Before the construction of radiomics model, we evaluated the reproducibility of image segmentation and influence of normalization in features generation, because there were concerns on the variability of individual segmentation^15^ and necessity of normalization on ADC map^14^. The segmentation step was not regarded as a step to introduce variances based on the result that average DICE coefficient between 3 readers was high. Our result also showed that 55% of top 20 radiomic features was consistently extracted with different normalization methods, which was selected among total 100 radiomic features. Furthermore, these 20 radiomic features accounted for 40–60% of features in the final 8 radiomics models.

In our radiomics models, the most relevant imaging features among the top 10 relevant descriptors was GLCM-derived features and the second-most relevant imaging features were first-order feature. This result is in similar context with a previous study by Corino et al.^16^ regarding that the two most relevant features for differentiation of high grade malignant soft tissue tumor were original first-order feature and GLCM. GLCM is a transformed matrix for texture-analysis, which calculates the occurrence of different gray level voxel pairs in certain spatial relationship. The GLCM features is known to reflect the tumor heterogeneity^17^, which is also a pathologically critical feature for histologic grade of soft tissue sarcoma^18,19^. Our result showed that radiomics using GLCM-derived features could be a measure to use quantitative information on tumor heterogeneity. However, radiomics models did not show superior diagnostic performance even with ADC combined. We assume that heterogeneity and cellularity might be parallel in soft tissue tumors. Therefore, radiomics signifying tissue heterogeneity could only provide redundant information to ADC. In addition, the soft tissue tumor consists of diverse histologic subtypes^13,20^ compared to other tumors. Therefore, it could be more difficult to find a universally effective radiomics model for all kinds of STTs rather than the model for specific tumor types. In other tumors composed histologically homogeneous cell type such as cervical cancer^21^ and prostate cancer^22^, the radiomics models presented similar or higher diagnostic performance compared to the diagnosis based on conventional diagnosis or ADC values.

Our study has several limitations. First, it is a retrospective study based on a single center with uniform MR protocol. As we divided training set and test set with temporal separation, by setting the patients who took MRI later to test set, there could be result interruption due to heterogeneity between two sets. Also, although we performed ten-fold cross validation to monitor and tune the model during training phase and subsequently tested the trained model with temporally split sample, external validation with data set from different institution is regarded as optimal way to prove the generalizability. Second, a certain type of tumor consists more than 50% of benign tumor groups in our study. In case of schwannoma, it is relatively straightforward to be diagnosed as benign with conventional images. However, this composition reflects the real incidence in clinical practice. It is still unclear whether balanced composition is beneficial to train model than the composition reflecting real incidence. Third, there are several concerns yet to be addressed in radiomics research such as stability of radiomic feature extraction, and difficulty in correlation with biological behaviors of targeted disease. Although many researches are ongoing to address these issues in radiomics, there is not sufficient evidence to support the radiomics as more robust and explainable methodology.

In conclusion, our study showed both ADC measurement and radiomics approach for ADC map are comparable for differentiating malignant and benign STT. However, we did not find additional diagnostic values of radiomics approach to conventional ADC measurement. Further study with a larger cohort from multiple institutions should ensue to prove the incremental values of radiomics approach.

## Material and methods

This retrospective study was approved by our institutional review board (IRB of The Catholic university of Korea, Seoul St. Mary’s Hospital) and the requirement for informed consent was waived. All methods in our study were carried out in accordance with relevant guidelines and regulations.

### Patient population

**Figure 3:**
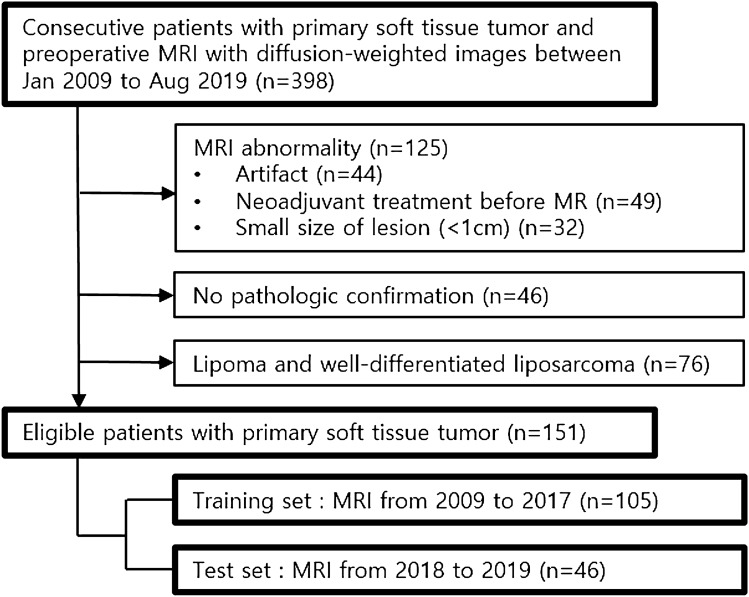
Flowchart of patient inclusion.

From January 2009 to August 2019, a total of 398 patients underwent 3.0 T MRI including DWI in our institution for primary soft tissue tumor evaluation. The MR images of 125 patients were excluded for various reasons: distortion of images due to artifacts (n = 44), acquisition of images after treatment (n = 49) and images of less than 1 cm sized lesions (n = 32). We also excluded well-differentiated adipocytic tumors (n = 76) such as lipomas and well-differentiated liposarcomas because DWI was performed using a single-shot, spin-echo echo-planar imaging sequence with fat suppression^1^. After excluding 46 additional patients who had not achieved pathologic confirmation, 151 patients were finally included (Fig. 3). All tumors were pathologically confirmed by surgical excision with histological analysis on the excisional sample performed by one pathologist. The specific histologic results and locations of soft tissue tumors were evaluated. The time intervals between MRI and pathologic result were additionally assessed. Among 151 data sets from 151 patients, 105 data sets were assigned to the training set. Forty-six consecutive patients who received MRI recently between 2018 and 2019 were assigned to the test set for temporal validation^23^.

### MRI protocols

MRI was obtained before surgery or neoadjuvant treatment in all patients. MRI was performed using two 3.0 T imagers (Verio and Magnetom Vida; Siemens Medical Solutions, Erlangen, Germany) with dedicated surface coils depending on the location of tumor. The standard MRI protocols included longitudinal fat-suppressed T2-weighted turbo spin-echo (TSE) sequence, axial T1-weighted TSE sequence, axial T2-weighted TSE sequences with and without fat suppression, and longitudinal and axial fat-suppressed contrast-enhanced T1-weighted TSE sequences. Other parameters are shown in Supplementary Table S2. Before contrast enhancement, a single-shot spin-echo echo-planar DWI sequence was obtained on the axial plane. A parallel imaging technique using GRAPPA (GeneRalized Autocalibrating Partially Parallel Acquisitions) was combined with an acceleration factor of 2. Sensitizing diffusion gradients were applied with b values of 0 and 800 s/mm^2^ sequentially in the x, y, and z directions^7^. Pixel-based ADC maps were created from DWI based on mono-exponential calculation using commercial software and a workstation (Leonardo MR Workplace; Siemens Medical Solution, Erlangen, Germany).

### MRI analysis

- ADC value acquisition
  In quantitative analysis of ADC, the ADC_mean_ and ADC_min_ values were measured by two readers (12-years and 2-years of experience in musculoskeletal radiology) on picture archiving and communication system (PACS)^24^. A solid portion for ROI setting was defined as the lesion showing hyperintense signal on DWI (b = 800 s/mm^2^), and enhancement on contrast enhanced T1-weighted images. Sites of hemorrhage, necrosis, or calcification were carefully avoided after correlation with standard MRI. After selection of solid portion, ROI was manually drawn on the ADC map, and the minimum and average values of the measurement were recorded as ADC_min_ and ADC_mean_. The acquired values from test set were used as a standard reference for evaluating the diagnostic performance of radiomics models.

### Radiomics model development and validation

**Figure 4:**
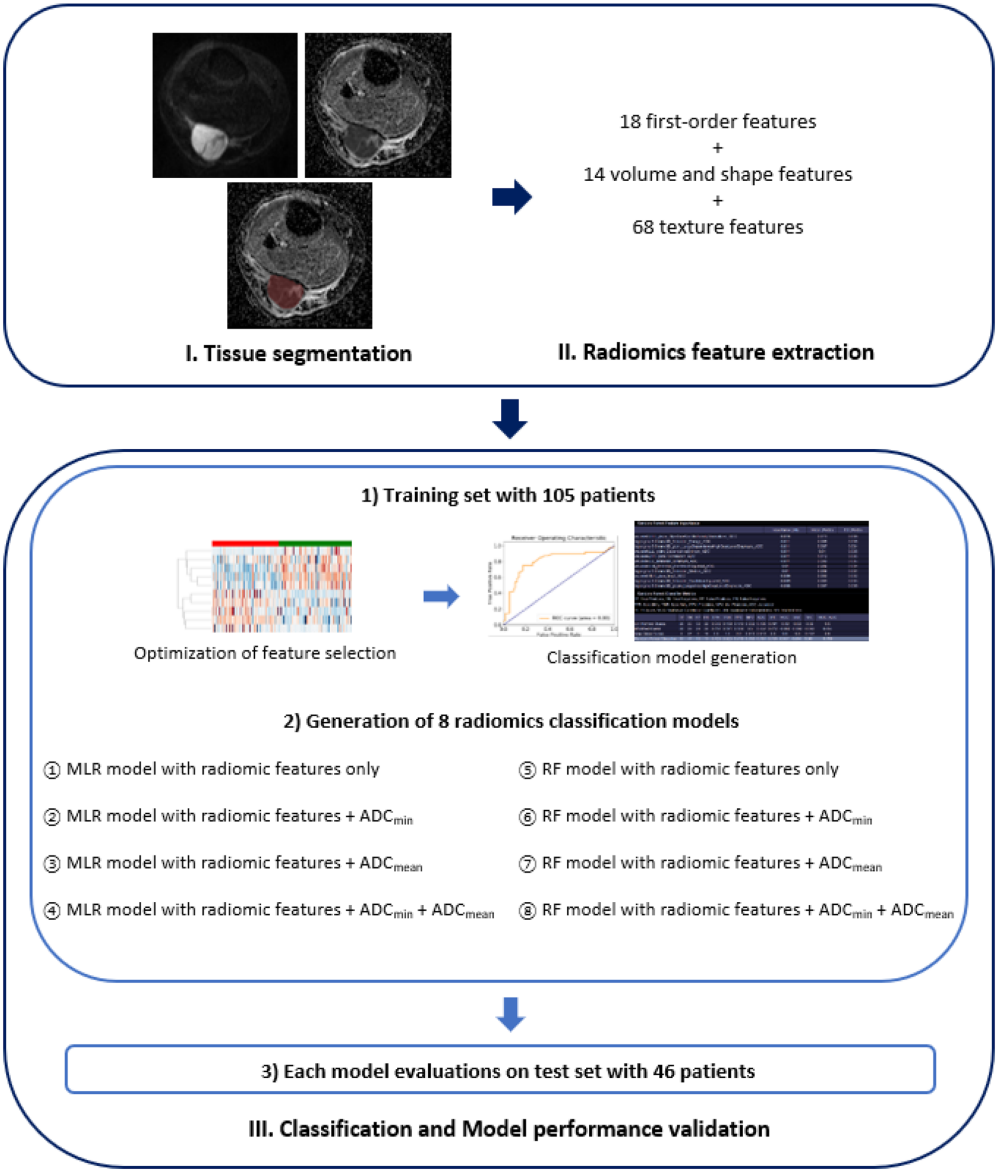
Radiomics pipeline of our study. The diagram was drawn by the first author of the manuscript. The images included in the diagram were captured from the softwares we used : ITK-SNAP software, version 3.8.0 (open source, http://itksnap.org/), Syngo. via Frontier Radiomics (Siemens Healthineers).

- Segmentation
  Segmentation was initially performed by another radiologist (2-years of experience on musculoskeletal radiology) using semiautomatic region intensity filter method, which was implemented by ITK-SNAP software, version 3.8.0 (open source, http://www.itksnap.org/)^25^. The segmented masks were manually revised on the b value image of 800 s/mm^2^ and co-registered ADC map with standard MRI as reference. VOI was drawn along the entire mass except for the most peripheral portions in order to avoid partial-volume effects. To review the reproducibility of VOI segmentation, the final correction of peripheral portion in VOI was edited in 20 tumor images by three readers, consisted of one student and two radiologists (2-years of experience on musculoskeletal radiology, each).
- Image preprocessing and radiomic feature extraction
  The single VOI was selected by consensus of three readers for further preprocessing steps. After VOI confirmation, the normalization of ADC map was done with the Z-score normalization, according to the following equation: $f\left( x \right) = \frac{{s\left( {x - \mu_{x} } \right)}}{{\sigma_{x} }}$ , with $f\left( x \right)$ as normalized intensity, $x$ as original intensity, $\mu_{x}$ as mean and $\sigma_{x}$ as standard deviation of image signal intensity, respectively^26^. According to Schwier et al.^14^, the image normalization provides the reproducibility of ADC map extracted radiomics features. In our study, normalization was performed using three different image coverages: (1) coverage of all voxels from image, including both VOI and background area (whole-image method), (2) coverage of voxels from VOI only (VOI method), (3) coverage of voxels from VOI with marginal dilation of 2 mm (VOI-dilation method), for inclusion of surrounding normal tissues. In this step, we assessed the influence of different normalization methods on the radiomic feature selection. The gray-level quantization and voxel resampling were performed with bin width of 5, and a spatial resolution of 3 × 3 × 3 mm^3^ using spline interpolator. Radiomic feature were extracted by using the pyradiomics package (https://github.com/Radiomics/pyradiomics/)^26^. Within each VOI, (a) 18 first-order features, (b) 14 volume and shape features, and (c) 68 texture features were obtained.
- Feature selection and classification model building
  We used Syngo. via Frontier Radiomics (Siemens Healthineers)^27^ to construct classification model. This software incorporates mRMR for feature selection, and multivariate logistic regression test and random forests (RF) algorithm for classification model algorithms, respectively. Classic mRMR is frequently used feature selection method for the exclusion of redundant features^28^, and applied to the selection of radiomic features for generating 8 radiomics models in our study. RFs for classification is one of the well-established classifiers in radiomics by constructing a multitude of decision trees^27,29–31^.
  In training phase, 10 radiomic features were selected by classic mRMR. Subsequently, classification models were developed using multivariate logistic regression (MLR) and random forest (RF). Each model was trained with 4 different inputs using radiomic features only, radiomic features and ADC_min_ combined, radiomic features and ADC_mean_ combined, and radiomic features and ADC_min_, ADC_mean_ combined. As a result, 8 classification models were built: (1) multivariate logistic regression (MLR) with radiomic features (MLR-R), radiomic features and ADC_min_ (MLR-I), radiomic features and ADC_mean_ (MLR-E), and radiomic features and ADC_min_, ADC_mean_ (MLR-A), (2) random forest (RF) with radiomic features (RF-R), radiomic features and ADC_min_ (RF-I), radiomic features and ADC_mean_ (RF-E), and radiomic features and ADC_min_, ADC_mean_ (RF-A). The best subset of features in MLR are determined with forward selection method using *adjusted R*^2^.
  Hyperparameters of RF used in the software package were summarized in supplementary material (S3). Accuracy, sensitivity, specificity, and area under the receiver operating characteristic curve (AUC) of each model was calculated in training set. In case of four RF models, averaged AUCs with ten-fold cross validation were calculated. Ten-fold cross validation is a resampling technique by dividing the data sample into 10 parts, and using 9 parts for training and 1 part for testing. After repeating the model training and validating procedure for 10 times with changing of training set sequentially, the model performance is determined by averaging all results acquired from 10 times of test set validation. Ten-fold cross validation process can protect the model against the overfitting, and overall error estimate is generalized.
- Temporal validation with test set
- Eight constructed classifier models were evaluated on a test set, which is temporally separated from the training set. Diagnostic accuracy, sensitivity, specificity, and AUC for differentiating malignant from benign soft tissue tumors were calculated. Entire step for radiomics workflow is demonstrated on Fig. 4.

### Statistical analysis

Student’s t-test and chi-square test were used to assess the difference between the training and test sets regarding the demographic data. Interobserver agreement of ADC_mean_ and ADC_min_ between two readers were evaluated using intraclass correlation (ICC) analysis. The sensitivity, specificity and AUC of ADC_mean_ and ADC_min_ of two readers were calculated in training set, respectively. The sensitivity and specificity were determined by selecting the optimal cut-off values as the minimum distance from the left upper corner of the unit square in ROC curves of ADCs.

During the image preprocessing step, DICE coefficient was calculated to measure the similarity between segmentations drawn by 3 readers. For the assessment of influence of normalization method on radiomic feature selection, the univariate regression test using Benjamini–Hochberg procedure with false positive rate of 0.05 was performed to extract 3 different sets of top 20 radiomic features, selected from 3 different normalization methods.

To compare the diagnostic performance between ADC measured on single layer and newly developed eight models including radiomic features, the ROC curves of ADC_mean_, ADC_min_, MLR-R, RF-R, MLR-I, RF-I, MLR-E, RF-E, MLR-A and RF-A models in test set were compared using the Delong test. All statistical analyses were performed using R version 4.0.0 (http://www.r-project.org/) and MedCalc for Windows, version 19.0 (MedCalc Software, Ostend, Belgium). A *p* value of < 0.05 was considered statistically significant.

## Supplementary Information

Supplementary Information.

## Data Availability

The datasets generated during and/or analyzed during the current study are available from the corresponding author on reasonable request.

## References

[1] Nagata S (2008). Diffusion-weighted imaging of soft tissue tumors: Usefulness of the apparent diffusion coefficient for differential diagnosis. Radiat. Med..

[2] Subhawong TK, Jacobs MA, Fayad LM (2014). Insights into quantitative diffusion-weighted MRI for musculoskeletal tumor imaging. AJR Am. J. Roentgenol..

[3] Lambin P (2012). Radiomics: extracting more information from medical images using advanced feature analysis. Eur. J. Cancer.

[4] Jeon JY, Chung HW, Lee MH, Lee SH, Shin MJ (2016). Usefulness of diffusion-weighted MR imaging for differentiating between benign and malignant superficial soft tissue tumours and tumour-like lesions. Br. J. Radiol..

[5] Lee SY (2016). Differentiation of malignant from benign soft tissue tumours: use of additive qualitative and quantitative diffusion-weighted MR imaging to standard MR imaging at 3.0 T. Eur. Radiol..

[6] Razek A, Nada N, Ghaniem M, Elkhamary S (2012). Assessment of soft tissue tumours of the extremities with diffusion echoplanar MR imaging. Radiol. Med..

[7] Khoo MM, Tyler PA, Saifuddin A, Padhani AR (2011). Diffusion-weighted imaging (DWI) in musculoskeletal MRI: a critical review. Skelet. Radiol..

[8] Lin Y-C (2017). Diffusion radiomics analysis of intratumoral heterogeneity in a murine prostate cancer model following radiotherapy: Pixelwise correlation with histology. J. Magn. Reson. Imaging.

[9] Pathak R (2017). A data-driven statistical model that estimates measurement uncertainty improves interpretation of ADC reproducibility: A multi-site study of liver metastases. Sci. Rep..

[10] Weller A (2017). Diffusion-weighted (DW) MRI in lung cancers: ADC test-retest repeatability. Eur. Radiol..

[11] Winfield JM (2015). Modelling DW-MRI data from primary and metastatic ovarian tumours. Eur. Radiol..

[12] Brynolfsson P (2017). Haralick texture features from apparent diffusion coefficient (ADC) MRI images depend on imaging and pre-processing parameters. Sci. Rep..

[13] Peerlings J (2019). Stability of radiomics features in apparent diffusion coefficient maps from a multi-centre test-retest trial. Sci. Rep..

[14] Schwier M (2019). Repeatability of multiparametric prostate MRI radiomics features. Sci. Rep..

[15] Cattell R, Chen S, Huang C (2019). Robustness of radiomic features in magnetic resonance imaging: review and a phantom study. Vis. Comput. Ind. Biomed. Art.

[16] Corino VDA (2018). Radiomic analysis of soft tissues sarcomas can distinguish intermediate from high-grade lesions. J. Magn. Reson. Imaging.

[17] Materka, A. & Strzelecki, M. Texture analysis methods—A review. COST B11 Report (1998).

[18] Crombé A (2019). Soft-tissue sarcomas: Assessment of MRI features correlating with histologic grade and patient outcome. Radiology.

[19] Zhao F (2014). Can MR imaging be used to predict tumor grade in soft-tissue sarcoma?. Radiology.

[20] Hong JH (2019). Soft tissue sarcoma: adding diffusion-weighted imaging improves MR imaging evaluation of tumor margin infiltration. Eur. Radiol..

[21] Liu Y (2019). Radiomics analysis of apparent diffusion coefficient in cervical cancer: A preliminary study on histological grade evaluation. J. Magn. Reson. Imaging.

[22] Bonekamp D (2018). Radiomic machine learning for characterization of prostate lesions with MRI: Comparison to ADC values. Radiology.

[23] Park SH, Han K (2018). Methodologic guide for evaluating clinical performance and effect of artificial intelligence technology for medical diagnosis and prediction. Radiology.

[24] El Kady RM, Choudhary AK, Tappouni R (2011). Accuracy of apparent diffusion coefficient value measurement on PACS workstation: A comparative analysis. Am. J. Roentgenol..

[25] Yushkevich PA (2006). User-guided 3D active contour segmentation of anatomical structures: significantly improved efficiency and reliability. Neuroimage.

[26] van Griethuysen JJM (2017). Computational radiomics system to decode the radiographic phenotype. Cancer Res..

[27] Medical Imaging 2019. Computer-Aided Diagnosis, San Diego, California, United States, 16–21 February 2019 in *Book Medical Imaging 2019: Computer-Aided Diagnosis, San Diego, California, United States, 16–21 February 2019* (ed. Editor) (SPIE, 2019).

[28] Peng H, Long F, Ding C (2005). Feature selection based on mutual information: Criteria of max-dependency, max-relevance, and min-redundancy. IEEE Trans. Pattern Anal. Mach. Intell..

[29] Breiman L (2001). Random forests. Mach. Learn..

[30] Erickson BJ, Korfiatis P, Akkus Z, Kline TL (2017). Machine learning for medical imaging. Radiographics.

[31] Parmar C, Grossmann P, Bussink J, Lambin P, Aerts H (2015). Machine learning methods for quantitative radiomic biomarkers. Sci. Rep..

