# Supplementary material for: Radiomics of diffusion-weighted MRI compared to conventional measurement of apparent diffusion-coefficient for differentiation between benign and malignant soft tissue tumors
Source: Sci Rep. 2021 Jul 27;11:15276. doi: 10.1038/s41598-021-94826-w (PMC8316538; doi:10.1038/s41598-021-94826-w)
Supplement: Supplementary file 1 — Supplementary Information. [file 41598_2021_94826_MOESM1_ESM.pdf]

Radiomics of diffusion-weighted MRI compared to conventional measurement of apparent diffusion-coefficient for differentiation between benign and malignant soft tissue tumors

Seung Eun Lee[1], MD, *Joon-Yong Jung[1], MD, Yoonho Nam[2], PhD, So-Yeon Lee[1], MD, Hyerim Park[3], MD, Seung-Han Shin[4], MD, Yang-Guk Chung[4], MD, Chan-Kwon Jung[5], MD

S1. Supplementary Table 1. Baseline demographics and clinical characteristics of patients

		Training set (n=105)	Test set (n=46)	p-value
**Patients (malignant : benign)**		47 : 58	24 : 22	0.403
**Sex (M : F)**		38 : 67	19 : 27	0.552
**Age (years, mean±SD)**		54.8±16.9	52.2±17.5	0.403
**ADC_{mean} (mean±SD, μm^2/sec)**	**Reader 1**	1448.59±567.98	1419±421.07	0.732
	**Reader 2**	1429.26±554.75	1424.82±448.91	0.962
**ADC_{min} (mean±SD, μm^2/sec)**	**Reader 1**	1015.55±492.67	980.35±375.26	0.635
	**Reader 2**	959.07±480.27	992.28±378.03	0.680

S2. Supplementary Table 2. MRI parameters in standard and diffusion-weighted imaging sequences

Parameters	Standard sequences	DWI (single shot)
**Field of view**	80-300mm	80-300mm
**Matrix size**	128x61 – 512 x 358	98x84 – 150x136
**TR (msec)/TE (msec)**	T1WI : 700-800/11-17<br>T2WI : 3000-5500/53-88	3500-7500/50-63
**Fat suppression**	DIXON	SPAIR
**Section thickness**	3-7	3-7
**Intersection gap**	0-0.6	0-0.6
**Turbo factor or EPI factor**	T1WI : 3<br>T2WI : 17	31-61
**Number of excitation**	1	3-8

S3. Hyperparameters in Random Forest Classifier implementation

based on scikit-learn :

(https://scikit-learn.org/stable/modules/generated/sklearn.ensemble.RandomForestClassifier.html)

The parameters are used:

- Number of trees in the forest: 100 (user can specify the number of trees in the prototype)
- Function to measure the quality of a split: Gini impurity
- Maximum depth: None
- Minimum number of samples required to split an internal node: 2
- Minimum number of samples required to be at a leaf node: 1
- Minimum weighted fraction of the sum of total weights (of all the input samples) required to be at a leaf node: 0
- Samples have equal weight
- Number of features to consider when looking for the best split: sqrt(numFeatures) *none if preselected features were used as input)
- Unlimited number of leaf nodes
- Min_impurity_decrease = 0
- Min_impurity_split = 0
- Bootstrap = True
- Use out-of-bag samples to estimate generalization accuracy: False
